# Early adulthood socioeconomic trajectories contribute to inequalities in adult cardiovascular health, independently of childhood and adulthood socioeconomic position

**DOI:** 10.1136/jech-2021-216611

**Published:** 2021-08-06

**Authors:** Eleanor M Winpenny, Laura D Howe, Esther M F van Sluijs, Rebecca Hardy, Kate Tilling

**Affiliations:** 1 MRC Epidemiology Unit, University of Cambridge, Cambridge, UK; 2 MRC Integrative Epidemiology Unit, University of Bristol, Bristol, UK; 3 CLOSER, Social Research Institute, UCL, London, UK

**Keywords:** inequalities, blood pressure, cardiovascular diseases, life course epidemiology, social epidemiology

## Abstract

**Background:**

Cardiovascular health shows significant socioeconomic inequalities, however there is little understanding of the role of early adulthood in generation of these inequalities. We assessed the contribution of socioeconomic trajectories during early adulthood (16–24 years) to cardiovascular health in mid-adulthood (46 years).

**Methods:**

Participants from the 1970 British Cohort Study with socioeconomic data available in early adulthood were included (n=12 423). Longitudinal latent class analysis identified socioeconomic trajectories, based on patterns of economic activity throughout early adulthood. Cardiometabolic risk factors (46 years) were regressed on socioeconomic trajectory class (16–24 years), testing mediation by adult socioeconomic position (46 years). Models were stratified by sex and adjusted for childhood socioeconomic position (SEP) and adolescent health.

**Results:**

Six early adulthood socioeconomic trajectories were identified: (1) Continued Education (20.2%), (2) Managerial Employment (16.0%), (3) Skilled Non-manual Employment (20.9%), (4) Skilled Manual Employment (18.9%), (5) Partly Skilled Employment (15.8%) and (6) Economically Inactive (8.1%). The ‘Continued Education’ trajectory class showed the best cardiovascular health at age 46 years, with the lowest levels of cardiometabolic risk factors. For example, systolic blood pressure was 128.9 mm Hg (95% CI 127.8 to 130.0) among men in the ‘Continued Education’ class, compared with 131.3 mm Hg (95% CI 130.4 to 132.2) among men in the ‘Skilled Manual’ class. Patterns across classes 2–6 differed by risk factor and sex. The observed associations were largely not mediated by SEP at age 46 years.

**Conclusion:**

Findings suggest an independent contribution of early adulthood socioeconomic trajectories to development of later life cardiovascular inequalities. Further work is needed to understand mediators of this relationship and potential for interventions to mitigate these pathways.

## Introduction

A large number of studies have shown that low socioeconomic position (SEP) in both childhood and adulthood is a risk factor for cardiovascular disease (CVD).^1–5^ Life course studies have suggested that the impacts of socioeconomic factors on cardiovascular outcomes may accumulate across the life course.^6–8^ However, the transitionary period of early adulthood (ages 16–24 years) has typically been overlooked despite it being considered a key time of social and economic transition where inequalities can emerge.^9^

Early adulthood is an important time for cardiovascular health.^10^ Life transitions which occur in early adulthood, for example, finishing education, starting employment or starting a family contribute to changes in key risk factors for later cardiovascular health, including lifestyle behaviours such as physical activity and diet.^11 12^ However, the transitory nature of early adulthood, when individuals may be moving in and out of education and short-term employment before settling into a longer term occupation, means that assessment of SEP at a single time point will not be reflective of experiences across the entire early adulthood period.

In this study, we investigate the contribution of early adulthood SEP to cardiovascular health in mid-adulthood, using data from the 1970 British Cohort Study (BCS70). We make use of longitudinal data on participation in education, employment/unemployment and social class of employment from age 16 to 24 years to generate socioeconomic trajectories across this early adulthood period. Our hypothesis is that the socioeconomic exposures of early adulthood contribute to cardiovascular outcomes independently of later adulthood SEP. To test this, we will address the following research questions:

1. What are the different socioeconomic trajectories followed across early adulthood (ages 16–24 years)?
2. What is the contribution of these trajectories to cardiovascular health in mid-adulthood (age 46 years)?
3. To what extent does SEP at age 46 years mediate this association?

## Methods

### Survey design and participants

The BCS70^13^ recruited ~17 000 people born in England, Scotland and Wales in a single week of 1970.^14^ Data on health, physical, educational, economic and social aspects of participants’ lives have been collected across nine sweeps: at birth and ages 5, 10, 16, 26, 30, 34, 38, 42 and 46 years. At age 46 years, 8581 cohort members participated in a nurse visit to collect anthropometric measures and blood samples for assessment of biomedical health. Written consent was obtained for blood sample collection and subsequent analysis and storage.^15^

### Exposure: early adulthood socioeconomic activity

Economic activity data were collected at ages 26, 30 and 34 years covering work and non-work activities since age 16 years. From these data, ‘activity histories’ have been developed which detail each episode of economic activity. We collapsed economic activity data from age 16 to 24 years into four main economic activity categories: education, employment, unemployment and economic inactivity, as shown in table 1, reflecting categories used by the UK Labour Force Survey.^16^ Those who were ‘economically inactive’ (ie, not in education, employment or seeking employment) were grouped together to distinguish these from those who were unemployed and seeking work, since the SEP of those economically inactive by choice is likely to be different from those looking for work. Of the episodes of economic activity classified as ‘economically inactive’, the majority (64%) were ‘looking after home/family’ and a further 19% ‘travelling/extended holiday’. Although these represent different exposures, we did not attempt to separate these groups due to the small numbers involved. We then brought together data on economic activity and occupational social class (UK Registrar General’s Social Class 1991) to categorise each period of activity into one of nine categories (table 1). This method allowed us to incorporate two commonly used measures of SEP (occupational social class and length of participation in education) into a single measure that could be captured longitudinally. The activity that was conducted for the longest period of each year of age, from age 16 to 24 years inclusive, was used as the primary activity for that year.

**Table 1:** Categories of early adulthood economic activity used in the analysis, derived from reported economic activity categories and occupational social class

Early adulthood economic activity	Reported economic activity categories	Occupational social class (RGSC 1991)	Examples of occupational social class^37^
Education	Full-time education Part-time education Government-training schemes	n/a	n/a
Professional employment	Full-time employment Part-time employment Self-employed (full or part-time)	Professional occupations	National government administrators, scientists, doctors
Managerial employment		Managerial and technical occupations	General managers, police officers, bank managers, teachers, nurses
Skilled non-manual employment		Skilled non-manual occupations	Salespeople, restaurant/shop managers, clerks, secretaries
Skilled manual employment		Skilled manual occupations	Plumbers, carpenters, mechanics, bus drivers
Partly skilled employment		Partly skilled occupations	Waiters, market traders, farm workers
Unskilled employment		Unskilled occupations	Labourers, porters
Unemployed	Unemployed and seeking work	n/a	n/a
Economically inactive	Looking after home/family Travelling/extended holiday Sick/disabled Voluntary work Maternity leave Retired	n/a	n/a

n/a, not applicable; RGSC, Registrar General’s Social Class.

### Outcomes: cardiometabolic risk factors

We studied the following metabolic risk factors, measured at age 46 years: waist circumference, systolic blood pressure (SBP), diastolic blood pressure (DBP), blood triglycerides, high-density lipoprotein cholesterol (HDL-c), non-HDL-c, glycated haemoglobin and C reactive protein (CRP). Further details of risk factor measurement are provided in the online supplemental methods. Measures of HDL-c, triglycerides and CRP were log-transformed, as these variables were positively skewed.

10.1136/jech-2021-216611.supp1 Supplementary data

### Covariates and descriptive variables

Two groups of covariates (potential confounders) were included in the analysis: (1) childhood SEP (parental social class, family income, parental education and family structure at age 10 years) and (2) adolescent health (parental report of health, body mass index (BMI) and malaise score at age 16 years). Adolescent health was considered a potential confounder since it may causally influence both the exposure and outcome. Adolescent health may also be driven by childhood SEP, hence adjustment for adolescent health may assist adjustment for childhood SEP. We additionally adjusted outcome measures for relevant medications taken at age 46 years, and we report on partnership and parenthood during early adulthood as descriptive variables for each early adulthood socioeconomic trajectory class. Further details of all covariates are found in the online supplemental methods.

### Mediators: SEP at age 46 years

Two indicators of adult SEP were tested as mediators: social class and income. These were measured at age 46 years, but are conceptualised as representing SEP across mid-adulthood since SEP is likely to be fairly stable at this age. Occupational social class at age 46 years was derived from participant’s report of their current employment, based on the UK National Statistics Socio-economic Classification (NS-SEC). Data were reported as eight categories: (1) higher managerial and administrative, (2) lower managerial and administrative, (3) intermediate occupations, (4) small employers and own account workers, (5) lower supervisory and technical, (6) semiroutine occupations, (7) routine occupations, (8) never worked and long-term unemployed.

Equivalised household income at age 46 years was calculated based on participant reporting of their household income and household composition. The weekly household income was divided by (1+0.5×number of additional adults+0.3×number of additional children (age 0–15 years)) based on the modified Organisation for Economic Co-operation and Development equivalence scale.^17^

### Statistical analysis

Longitudinal latent class analysis was conducted using Mplus, V.8.4, to generate classes of individuals based on their participation in different economic activities over time. All individuals who had economic activity data from any year from age 16 to 24 years were included (n=12 423). We added classes to the model sequentially, from one to eight classes, when the model failed to converge. The final number of latent classes used for the analysis was selected based on fit statistics (Akaike information criterion, Bayesian information criterion, Vuong-Lo-Mendell-Rubin likelihood ratio test, bootstrapped parametric likelihood ratio test) together with the interpretability of the classes generated.^18^ Missing data were addressed using full information maximum likelihood estimation. Given high classification accuracy (entropy of 0.97), most likely class membership for each individual was extracted from the latent class model and used as a categorical exposure for further analyses.

Descriptive analyses and regression models were performed using Stata, V.16. All individuals who had data on economic activity (n=12 423) were included in subsequent analyses. Associations between latent classes (representing early adulthood socioeconomic trajectories) and metabolic risk factors were tested using ordinary least squares linear regression. Analyses were conducted separately by sex, given differences in both class membership and health outcomes by sex. Missing covariate and outcome data were imputed by chained equations (under the missing at random assumption).^19 20^ We included as auxiliary variables SEP at birth, variables on mental and physical health at ages 10 and 16 years, and additional health variables from age 42 and 46 years, creating 20 imputed datasets (see online supplemental methods and table S1). We performed joint tests of coefficients across the socioeconomic trajectory classes to assess the overall effect of socioeconomic trajectory class on each of the outcome variables. The Stata ‘mimrgns’ command was used to calculate the average predicted value of each outcome for each socioeconomic trajectory class. To investigate the added value of the longitudinal SEP data, we also assessed associations between SEP measured at age 24 years only and metabolic risk factors, using the same imputation and covariates as in the main analysis.

**Figure 1:**
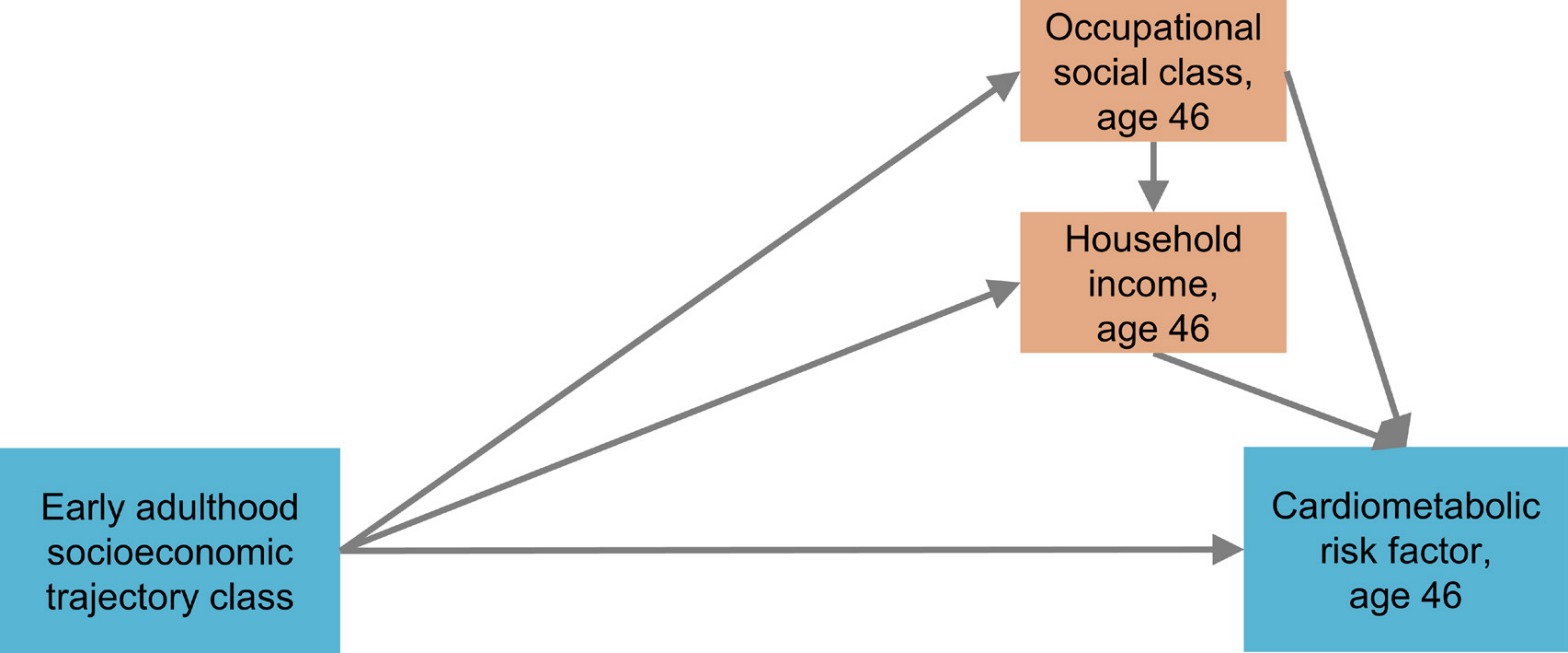
Path model of direct and indirect associations between early adulthood socioeconomic trajectory class and cardiometabolic health at age 46 years. Models were additionally adjusted for covariates measuring childhood socioeconomic position and adolescent health.

To examine mediation of associations by SEP at age 46 years, we used path models, in Mplus V.8.4. In addition to the direct regression path of cardiometabolic risk factors on socioeconomic trajectory class, we added two indirect paths from socioeconomic trajectory class to cardiometabolic risk factor, via occupational social class (NS-SEC), and via equivalised household income. A path from NS-SEC to income was also added to account for the likely contribution of occupational social class to household income (figure 1). Each outcome (cardiometabolic risk factor) was considered separately, adjusting for the same confounding factors as used in the regression analyses.

## Results

### Early adulthood socioeconomic trajectories

**Figure 2:**
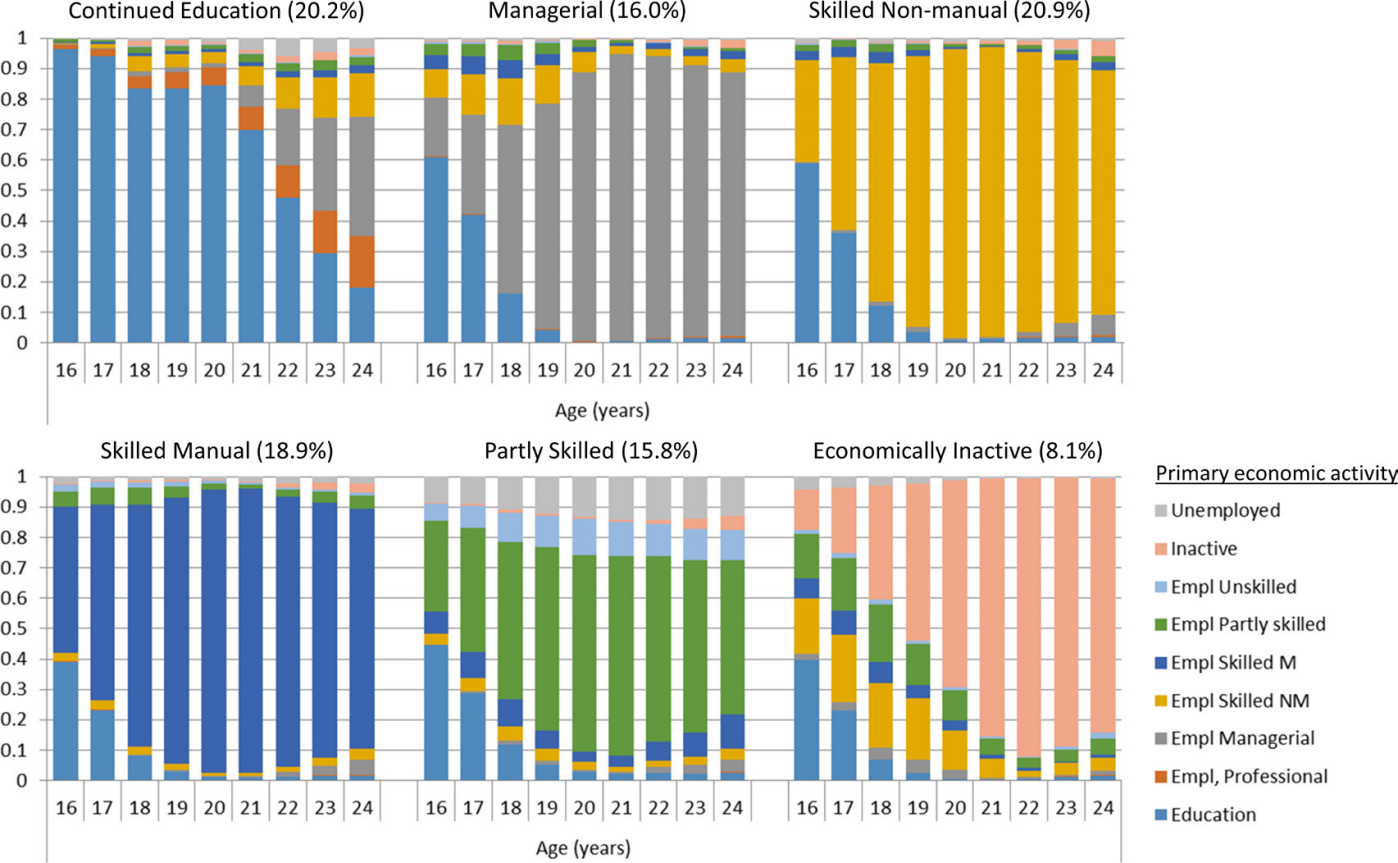
The six socioeconomic trajectory classes, showing response probabilities for participation in different economic activities at each year of age.

Fit statistics for the latent class analysis continued to improve with addition of classes, at a decreasing rate, up to seven classes, beyond which the model failed to converge (see online supplemental table S2). Examination of the response probabilities of participation in different economic activities in the 6 class and 7 class solutions (figure 2 and online supplemental figure S8) revealed that the 6 class solution was more interpretable, and this solution was therefore chosen for subsequent analysis. The 7 class solution (online supplemental figure S8) included similar classes to the 6 class, but added a small class (6%) which mixed people participating in professional employment, unskilled employment and unemployed. As shown in figure 2, the 6 class solution included a ‘Continued Education’ class, many of whom continued in education after age 18 years and entered professional, managerial or skilled non-manual occupations. Classes 2–5 were largely differentiated by the type of occupation conducted. Class 6 was the smallest group, who were primarily economically inactive towards the end of the early adulthood period. Entropy of the latent class model was high at 0.97, suggesting high classification accuracy. Comparison of membership of each socioeconomic trajectory class with assessment of their SEP at a single time point (age 24 years) is shown in online supplemental table S3. Although there is overall high correlation between these measures (Cramer’s V: 0.69), there are also differences based on the ability of the socioeconomic trajectory classes to capture longitudinal information across the early adulthood period. In particular, we find that the ‘Continued Education’ class includes those reporting professional, managerial or skilled non-manual employment at age 24 years. The ‘Partly Skilled’ class also combines those unemployed or in unskilled, partly skilled and skilled employment at age 24 years. In addition, those unemployed or with missing data at age 24 years are assigned to many different socioeconomic trajectory classes, based on their economic activity across the early adulthood period.

Table 2 reports differences in sociodemographic characteristics between the socioeconomic trajectory classes. Class 3 ‘Skilled Non-manual’ were more likely for women, while class 4 ‘Skilled Manual’ were more likely for men. Class 6 ‘Economically Inactive’ included primarily women who were partnered, and around 45% had children (table 2).

**Table 2:** Descriptive data by early adulthood socioeconomic trajectory class

		1. Continued Education (n=2515, 20.2%)	2. Managerial (n=1982, 16.0%)	3. Skilled Non-manual (n=2601, 20.9%)	4. Skilled Manual (n=2351, 18.9%)	5. Partly Skilled (n=1967, 15.8%)	6. Economically Inactive (n=1007, 8.1%)
Sex	Female (%)	46.8	48.3	74.9	21.7	40.1	90.6
Parental occupational social class, at participant age 10 years (RGSC) (n=10 365)	Professional (%)	16.4	5.5	4.4	2.2	2.6	1.5
	Managerial (%)	38.6	29.9	23.7	18.1	14.7	13.5
	Skilled non-manual (%)	11.7	13.7	13.1	9.1	7.3	9.1
	Skilled manual (%)	24.9	37.0	43.7	51.8	48.9	47.1
	Partly- skilled (%)	7.1	11.7	11.9	15.4	19.3	19.5
	Unskilled (%)	1.3	2.4	3.3	3.5	7.2	9.4
Partnered between age 16 and 24 years (n=12 423)	Had one or more partners (%)	42.6	58.8	61.6	58.0	55.8	78.4
Parent by 24 years (n=10 150)	Had one or more children (%)	2.3	8.1	8.6	13.7	16.3	45.2
Participant NS-SEC, age 46 years (n=6899)	Higher managerial, administrative and professional occupations (%)	38.9	21.2	15.2	9.2	6.3	4.0
	Lower managerial, administrative and professional occupations (%)	41.8	45.2	31.9	19.8	19.2	25.4
	Intermediate occupations (%)	6.6	9.4	28.4	7.6	9.0	17.4
	Small employers and own account workers (%)	5.4	8.6	6.4	17.5	12.2	5.4
	Lower supervisory and technical occupations (%)	2.3	5.6	3.9	22.3	15.0	7.7
	Semiroutine occupations (%)	2.5	5.4	9.1	9.4	20.2	26.5
	Routine occupations (%)	0.8	3.7	3.4	12.7	14.4	10.5
	Never worked and long-term unemployed (%)	1.7	0.9	1.7	1.5	3.5	3.1
Participant equivalised household income, age 46 years (n=7267)	£ per week mean (SD)	688 (1757)	623 (677)	446 (734)	439 (947)	338 (422)	277 (263)

Due to cohort attrition, the number of included participants is lower for age 46 variables (NS-SEC and equivalised household income).

NS-SEC, National Statistics Socio-economic Classification; RGSC, Registrar General’s Social Class.

### Associations between socioeconomic trajectory class and cardiometabolic risk factors

**Figure 3:**
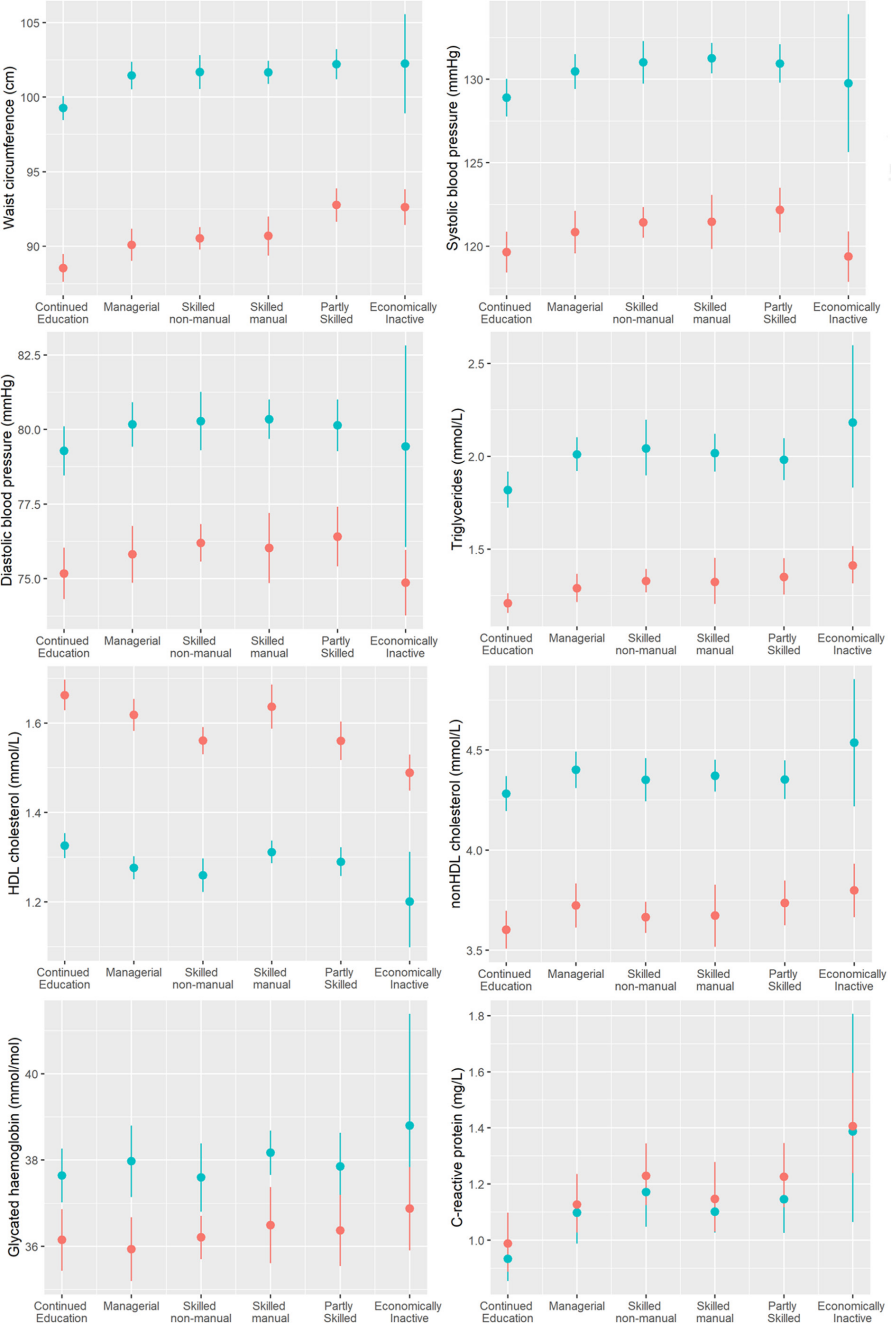
Modelled mean values (with 95% CIs) for each cardiometabolic outcome by early adulthood socioeconomic trajectory class. Red is female, blue is male. HDL, high-density lipoprotein.

Analysis showed differences in risk factors between socioeconomic trajectory classes for waist circumference, SBP, HDL-c, triglycerides and CRP. ‘Continued Education’ represented the healthiest class, with differing levels of risk factors seen between this class and the remaining classes across most outcomes. Cardiometabolic risk factors were less differentiated across the remaining socioeconomic trajectory classes, although patterns varied by outcome (figure 3). The ‘Economically Inactive’ class, although in general high risk across many of the outcomes, showed low SBP and DBP among women, comparable with the ‘Continued Education’ class. Figure 3 shows the estimated marginal means of each outcome by socioeconomic trajectory class (numerical data presented in online supplemental table S4).

For comparison, we report associations between cardiometabolic risk factors and SEP (based on economic activity and occupational social class) at a single time point at age 24 years (online supplemental table S5). The ‘Continued Education’ class cannot be identified using SEP measured at age 24 years, so the difference in risk factors between this class and the remaining classes cannot be observed. For those in managerial employment and skilled non-manual employment at age 24 years, slightly lower levels of risk factors are estimated than using the corresponding trajectory classes, likely because these SEP groups include some members who would be included in the ‘Continued Education’ class. Similarly, those who report being economically inactive at age 24 years show smaller associations with some cardiometabolic risk factors than those in the ‘Economically Inactive’ class, which captures prolonged economic inactivity.

### Mediation of associations by occupational social class and income at age 46 years

Investigating mediation of associations between early adulthood socioeconomic trajectories and cardiometabolic risk factors by occupational social class and income at age 46 years showed that for most outcomes tested, the indirect path via both of these mediators was small, with CIs that spanned the null (online supplemental table S6). The exception to this was for HDL-c where there was evidence of an indirect effect via NS-SEC at age 46 years, but this was in most classes of a lower magnitude than the direct effect (online supplemental table S6). Cross-sectional associations between occupational social class or income and cardiometabolic risk factors at age 46 years were much reduced after adjustment for early adulthood socioeconomic trajectories, as shown in online supplemental table S7.

## Discussion

### Main findings

This study identified six classes of individuals who follow different socioeconomic trajectories during early adulthood (ages 16–24 years). In our population, born in the UK in 1970, we found one highly educated class, four classes based primarily on occupational social class, and one class which was economically inactive. These early adulthood socioeconomic trajectory classes were associated with cardiometabolic risk factors in mid-adulthood, with the largest differences seen between the ‘Continued Education’ class and remaining classes. These associations were largely not mediated by SEP at age 46 years, suggesting an important independent role of the early adulthood period in determining cardiovascular inequalities in later life.

### Comparison with previously reported evidence

While a wide range of previous studies have reported associations between SEP and cardiovascular health,^1–3 21 22^ few have considered the contribution of the life stage of early adulthood. This is likely due in part to the difficulty of measuring SEP over this period, when individuals may be in education, in transient employment or economically inactive, often not yet settling into stable occupations.^23^ Comparable studies in other cohorts using a similar methodology to our own have not been conducted. Nevertheless, previous studies have shown associations between education level attained and metabolic syndrome^24 25^ or coronary heart disease.^26^ A study of the 1946 British Birth Cohort, which investigated relationships between education level, as well as childhood and adult social class, with metabolic syndrome, reported a contribution of educational inequalities in the development of metabolic syndrome, independent of childhood and adult social class.^26^ More recently, Mendelian randomisation studies have suggested a causal effect of educational attainment on BMI, SBP and CVD.^27 28^ These findings are consistent with our own findings regarding the independent role of early adulthood SEP for later cardiovascular health, and an important contribution of education in defining early adulthood SEP.

Further analysis of the 1946 British Birth Cohort, which included a binary measure of occupational social class at age 26 years as a marker of SEP in early adulthood, found that accumulation models which included early adulthood SEP together with childhood and middle age SEP provided the best fit for predicting cardiometabolic risk factors among women,^8^ and predicting measures of cardiac structure and function in both sexes.^29^ While these papers used a measure of occupational social class in the late 20s which lies outside the age range which we consider as early adulthood (ages 16–24 years), their findings are consistent with our own finding of an important role for young adult SEP.

We found that mid-adulthood social class contributed only marginally to inequalities in cardiometabolic risk factors, following adjustment for childhood and early adulthood social class. Although this may seem surprising, a study of the 1946 British Birth Cohort also found that associations between adult social class and the metabolic syndrome were attenuated below significance after adjustment for childhood social class and education level.^26^ In this study, we used a more comprehensive measure of early adulthood SEP which accounted for more than education level, therefore we might expect associations to be further attenuated than seen previously.

Overall, the size of the inequalities observed in cardiometabolic risk factors in this study are of considerable relevance for public health. For example, between the ‘Continued Education’ class and the ‘Partly Skilled’ class in men, we see a 3 cm difference in waist circumference, which has previously been associated with a 6% increase in the relative risk of a cardiovascular event,^30^ and a 2 mm Hg increase in SBP, associated with a 7% increased risk of vascular mortality.^31^

### Strengths and limitations

The strengths of this analysis lie in both the data and the methodology. The detailed data on economic activity reported by the BCS70 cohort allowed us to develop a new method to determine trajectories of early adulthood SEP. These trajectories incorporate education participation and occupational social class data collected across early adulthood, thereby providing a more comprehensive measure of early adulthood SEP which allows for non-linear as well as traditional pathways into adult work. This method also allows inclusion of all participants who contributed SEP data at any time point during early adulthood. The long follow-up of BCS70 allows examination of cardiometabolic risk factors much later in adulthood, likely to be highly relevant for cardiovascular morbidity and mortality. Nevertheless, our early adulthood trajectories necessarily represent the pathways followed by those born in 1970, which may differ from those that experienced by more contemporary early adulthood populations.

The BCS70 is a large birth cohort with a sampling frame designed to represent the population of Great Britain born in 1970. As with all birth studies there has been attrition over the course of the study, with differential attrition by sex and parental socioeconomic class,^32^ creating potential for introduction of bias. We included in our analysis all those who remained in the study during early adulthood, imputing missing outcome data and controlling for childhood covariates including those known to be associated with attrition. There is no reason to expect that participants lost to the study during childhood and adolescence differed in terms of their mid-adulthood cardiometabolic risk factors from those of similarly disadvantaged backgrounds who remained in the study until early adulthood.

There remains the possibility that our findings may be biased due to either measurement error or unmeasured confounding. Despite inclusion of several different measures of childhood SEP and adolescent health as covariates, it may be that there remains residual unmeasured confounding. There is also the possibility of unmeasured confounding of mediator-outcome associations in our mediation model, however given the small associations between mediators and outcomes, we judge this unlikely to introduce significant bias.

SEP is difficult to measure, making it possible that our exposure classes do not accurately reflect all aspects of SEP. Nevertheless, we believe that this study improves on previous studies which consider only a single measure of early adulthood SEP, for example, educational achievement or occupational social class at a single age, providing a more appropriate overview of SEP across the early adulthood period. The differences observed between associations using our socioeconomic trajectories and a single measure of SEP at age 24 years suggest that assessment of SEP across early adulthood is a more comprehensive measure, highlighting the importance of the ‘Continued Education’ class, and avoiding misclassification based on measurement of status at a single time point.

To generate the socioeconomic trajectory classes, we incorporated education based on educational participation over time, but were not able to include the level of education undertaken, since these data were not available. We therefore assume that for most participants, educational participation at older ages will correspond to participation at a higher level. We have also focused on participation in education and employment, and occupational class, and have grouped together those who were economically inactive for any reason other than education. In this analysis, we are therefore not able to distinguish between those who are looking after home or family, travelling, or unable to work through sickness or disability, and there may therefore be subgroups within the ‘Economically Inactive’ class who would show different associations with the cardiometabolic risk factors.

### Interpretation of findings

**Figure 4:**
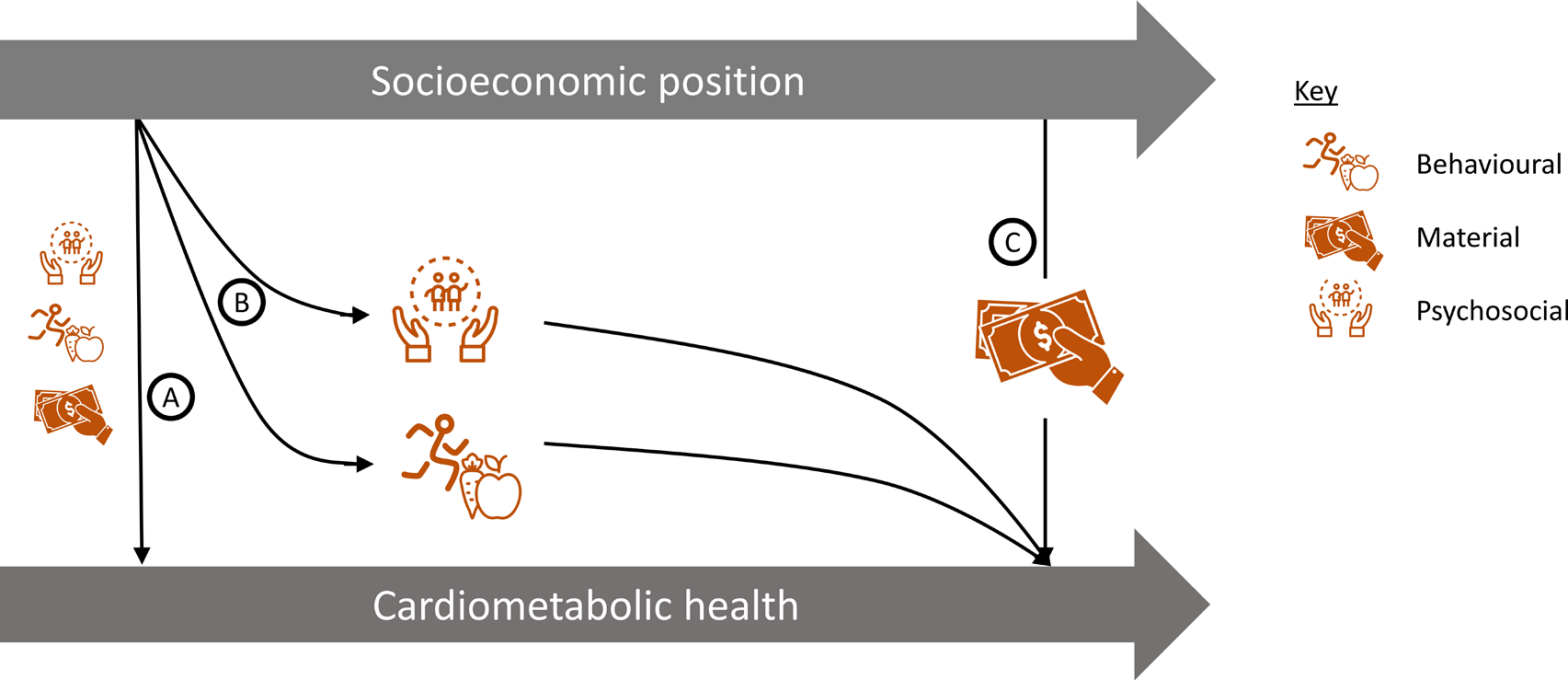
Potential pathways from early adulthood socioeconomic position to cardiometabolic health. (A) Short-term pathways to cardiometabolic health, (B) pathways mediated by development of behaviours and psychosocial factors in early adulthood, (C) material pathways in mid-adulthood.

Socioeconomic inequalities in health have been suggested to act through either (1) behavioural, (2) material or (3) psychosocial pathways.^33^ Short-term associations between early adulthood SEP and cardiovascular health may act through any of these three pathways (arrow A in figure 4), and are supported by recent research from the Cardiovascular Risk in Young Finns Study which reports a short-term association between young adults SEP with carotid intima-media thickness, a marker of subclinical atherosclerosis.^34^ In addition, since early adulthood is known to be an important time for development of health-related behaviours^35 36^ and psychosocial factors,^23^ early adulthood SEP may contribute to development of health behaviours or psychosocial factors, which then track through adulthood and have ongoing long-term effects on cardiometabolic risk factors. Our finding that early adulthood SEP is not mediated by SEP in mid-adulthood suggests that material factors in mid-adulthood do not form part of the pathway by which early adulthood SEP might contribute to mid-life cardiovascular inequalities (pathway C in figure 4). Further research will be needed to understand the contribution of pathways A and B depicted in figure 4.

### Conclusions and implications of the findings

Our research suggests that early adulthood is an important period for the generation of inequalities in later life cardiovascular health. This suggests a need for further study of this developmental stage of early adulthood to support health policy and interventions which aim to improve cardiovascular health and reduce inequalities. Despite the difficulty of measuring SEP in early adulthood, we provide a method for identifying early adulthood SEP which may be adapted for use in different cohorts. Our finding that early adulthood SEP is not mediated by measures of SEP in mid-adulthood suggests that other pathways are involved, with further research needed to understand and delineate these possible pathways.

**What is already known on this subject**

- Socioeconomic inequalities are a key contributor to differences in cardiovascular health, with the impact of socioeconomic inequalities thought to accumulate across the life course. We know that early adulthood is an important time for both the development of adult socioeconomic position (educational attainment and entering the job market) and for development of behaviours related to cardiovascular health. However, previous life course studies of cardiovascular health inequalities have not included a measure of socioeconomic position covering the transitional early adulthood period.

**What this study adds**

- This study develops a new method to assess socioeconomic position across the early adulthood period, and relates this to mid-life cardiovascular health. The study shows that the early adulthood period is an important period for development of cardiovascular health inequalities, largely driving the cross-sectional associations between socioeconomic position and cardiovascular risk factors observed in mid-life. This suggests that further intervention during the early adulthood period is needed to prevent development of inequalities in cardiovascular health in later life.

## Data Availability

Data are available in a public, open access repository. The data underlying this article are freely available to bona fide researchers via the UK Data Service (http://ukdataservice.ac.uk).
