# Supplementary material for: Early adulthood socioeconomic trajectories contribute to inequalities in adult cardiovascular health, independently of childhood and adulthood socioeconomic position
Source: J Epidemiol Community Health. 2021 Aug 6;75(12):1172–80. doi: 10.1136/jech-2021-216611 (PMC8588297; doi:10.1136/jech-2021-216611)
Supplement: Supplementary data [file jech-2021-216611supp001.pdf]

# Supplementary information

## Supplemental Methods

**Metabolic risk factors: further details of measures used**

Waist circumference was measured at the midpoint between the iliac crest and the lower rib (to the nearest 0·1 cm) using a tape measure. Systolic and diastolic blood pressures were measured in a seated position after participants had not eaten, consumed alcohol, smoked or participated in vigorous exercise in the preceding 30 min, using an Omron HEM 907 blood pressure monitor. Non-fasted blood samples were taken and processed to measure triglycerides, high-density lipoprotein cholesterol (HDL-c), total cholesterol, HbA1c and CRP, as described elsewhere.[37,38] Non-HDL-c was calculated by subtracting HDL-c from total cholesterol. A lower number of participants had data available on trigylcerides and CRP than the other outcome measures since only blood samples collected from mid-way through fieldwork (May 2017) were analysed for these outcomes.[38] We excluded those with values of CRP above 10mg/L from CRP analyses, as this is likely to reflect recent infection rather than chronic inflammation.

**Covariates and descriptive variables: further details of measures used**

Parental social class was based on father's occupation (or mother's if father's was missing) measured using the UK Registrar General's Social Class (RGSC) (6 categories) at age 10. Family income was reported as gross weekly family income (7 categories) at age 10. Data on maternal and paternal education was recoded into 5 categories: No qualifications, Trade apprenticeship/O-level, A-level, Further vocational, Degree. Parental education was taken as the higher level of education reported from either the father or the mother. Family structure was derived from questions which asked the relationship to the participant of the 'mother figure' and 'father figure' that participants were living with at age 10. This was reduced to four categories: "2 natural parents", "one parent and one other", "single parent alone", "no natural parents".

Adolescent health was measured at age 16. Malaise score was calculated from the 24-item self-completion Malaise Inventory. A parental questionnaire item asked about the participant's health over the past 12 months, responses were dichotomised to good or poor health. Height (in meters) and weight (in kilograms) were measured at age 16 by a community medical officer or school nurse, and used to calculate BMI (kg/m2).

Data on partnership was included from the BCS70 partnership histories which combine data on periods of partnership from across multiple waves of data collection. We generated a binary variable which indicated whether the individual had had a partner (cohabitation or marriage) at any time between the ages of 16 and 24y. We used data on whether the participant or their partner had been pregnant, collected at age 29, together with the date of birth of the first child to determine whether the participant had had a child by the age of 24y.

Medication data was collected at age 46y and coded by the nurses to sub-chapter level of the British National Formulary edition 69. To account for the effect of medications on metabolic risk factors, we added a constant of 10mmHg and 5mmHg to measures of SBP and DBP respectively, among those taking medications for hypertension (n=495),.[39,40] We multiplied measures of triglycerides, HDL-c and total cholesterol by 1.25, 0.96 and 1.5 respectively, among those reporting taking lipid-regulating drugs (n=257), to adjust for the treatment effect of atorvastatin, the most frequently prescribed lipid-regulating drug in the UK.[41] Measures of HbA1c were multiplied by 1.01 where participants reported taking diabetes medications (n=185).[42] Analyses of HbA1c were additionally included as covariates: antiplatelet drugs, anti-inflammatory medications which may influence HbA1c level, while analyses of CRP additionally included as covariates: anti-inflammatory medications, lipid-regulating drugs, hormone medications and contraceptive medications.[43]

**Statistical analysis: imputation**



Winpenny EM, *et al. J Epidemiol Community Health* 2021; 75:1172–1180. doi: 10.1136/jech-2021-216611

For all outcomes, except for C-reactive protein, a single imputation model was used which included the exposure, all outcomes and covariates, and auxiliary variables, as shown in TableS4. Missing values were imputed using chained equations, using the Stata 'mi impute chained' command. Ordered logistic regression was used for ordinal variables and linear regression for continuous variables, creating 20 imputed datasets. C-reactive protein was imputed separately by predictive mean matching, after participants with values of CRP >10mg/L had been excluded, but including the same variables in the imputation model, as described in Table S1.

**Table S1: Variables used in multiple imputation by chained equations.**

| Variable | Type of variable | Model used to predict missing data in this variable | N (%) with data on this variable |
|---|---|---|---|
| **Outcome variables** | | | |
| Waist circumference, age 46 | Continuous | Linear regression | 6993 (56%) |
| SBP, age 46 | Continuous | Linear regression | 7061 (57%) |
| DBP, age 46 | Continuous | Linear regression | 7060 (57%) |
| logHDL cholesterol, age 46 | Continuous | Linear regression | 5654 (46%) |
| Non-HDL cholesterol, age 46 | Continuous | Linear regression | 5653 (46%) |
| Log Triglycerides, age 46 | Continuous | Linear regression | 3194 (26%) |
| HbA1c, age 46 | Continuous | Linear regression | 5613 (45%) |
| CRP, age 46 | Continuous | Predictive mean matching | |
| **Mediators** | | | |
| NS-SEC8, age 46 | Ordered categorical | Ordered logistic regression | 6899 (56%) |
| Equivalised income, age 46 | Continuous | Linear regression | 7267 (58%) |
| **Exposure variable** | | | |
| Early adulthood socioeconomic trajectory class | Ordered categorical | n/a | 12423 (100%) |
| **Model covariates** | | | |
| Sex | Binary | n/a | 12423 (100%) |
| Parental social class, age 10 | Ordered categorical | Ordered logistic regression | 10365 (83%) |
| Parental education, age 10 | Ordered categorical | Ordered logistic regression | 10517 (85%) |
| Parental income, age 10 | Ordered categorical | Ordered logistic regression | 9846 (79%) |
| Family structure, age 10 | Ordered categorical | Ordered logistic regression | 10709 (86%) |
| Malaise scale, age 16 | Continuous | Linear regression | 4760 (38%) |
| Poor health, age 16 | Ordered categorical | Ordered logistic regression | 7052 (57%) |
| BMI, age 16 | Continuous | Linear regression | 4975 (40%) |
| **Auxiliary variables for outcomes** | | | |
| BMI, age 42 | Continuous | Linear regression | 8240 (66%) |
| Warwick Edinburgh Mental Well being scale, age 42 | Continuous | Linear regression | 7596 (61%) |
| Malaise Inventory score, age 42 | Ordered categorical | Ordered logistic regression | 8056 (65%) |
| Long-standing limiting illness, age 42 | Ordered categorical | Ordered logistic regression | 9143 (74%) |
| Self-assessed general health, age 42 | Ordered categorical | Ordered logistic regression | 9113 (73%) |
| **Auxiliary variables for mediators** | | | |
| NSSEC8, age 42 | Ordered categorical | Ordered logistic regression | 8006 (64%) |
| Cohort member and partner's take home income, age 42 | Ordered categorical | Ordered logistic regression | 6278 (51%) |
| **Auxiliary variables for age 16 health covariates** | | | |

| | | | |
|---|---|---|---|
| Weight, age 16 | Continuous | Linear regression | 5104 (41%) |
| SBP, age 16 | Continuous | Linear regression | 5089 (41%) |
| DBP, age 16 | Continuous | Linear regression | 5079 (41%) |
| BMI, age 10 | Continuous | Linear regression | 9606 (77%) |
| SBP, age 10 | Continuous | Linear regression | 10128 (82%) |
| DBP, age 10 | Continuous | Linear regression | 10128 (82%) |
| Rutter behaviour scale, age 10 | Ordered categorical | Ordered logistic regression | 10076 (81%) |
| Malaise Inventory score, age 29 | Continuous | Linear regression | 11170 (90%) |
| **Auxiliary variables for childhood SES covariates** | | | |
| Father's social class, birth | Ordered categorical | Ordered logistic regression | 10719 (86%) |
| Mother's social class, birth | Ordered categorical | Ordered logistic regression | 7059 (57%) |
| Father's age at completion of education, birth | Continuous | Linear regression | 11005 (89%) |
| Mother's age at completion of education, birth | Continuous | Linear regression | 11398 (92%) |
| Family structure, age 5 | Ordered categorical | Ordered logistic regression | 10150 (82%) |



Winpenny EM, *et al. J Epidemiol Community Health* 2021; 75:1172–1180. doi: 10.1136/jech-2021-216611

# Supplementary Results

**Table S2: Fit indices for latent class models, testing different numbers of latent classes.**

| Classes | AIC | BIC | aBIC | Entropy | VLMR_PValue | LMR_PValue | BLRT_PValue | min_N | min_prob |
|---|---|---|---|---|---|---|---|---|---|
| 2 | 322309.236 | 323386.195 | 322925.4 | 0.959 | 0.00 | 0.00 | 0.00 | 5191.405 | 0.41789 |
| 3 | 287735.194 | 289354.346 | 288661.565 | 0.96 | 0.00 | 0.00 | 0.00 | 3590.5 | 0.28902 |
| 4 | 264445.405 | 266606.751 | 265681.984 | 0.961 | 0.00 | 0.00 | 0.00 | 2477.8 | 0.19946 |
| 5 | 246497.232 | 249200.771 | 248044.018 | 0.961 | 0.00 | 0.00 | 0.00 | 1995 | 0.16057 |
| 6 | 235012.643 | 238258.375 | 236869.636 | 0.963 | 0.00 | 0.00 | 0.00 | 989 | 0.07962 |
| 7 | 227952.548 | 231740.474 | 230119.748 | 0.963 | 0.00 | 0.00 | 0.00 | 730 | 0.0588 |

**Table S3: Cross-tabulation of membership of socioeconomic trajectory classes with SEP based on economic activity and occupational social class at age 24y.**

| | **Socioeconomic trajectory class** | | | | | | |
|---|---|---|---|---|---|---|---|
| **Economic activity, age 24y** | **Continued education** | **Managerial** | **Skilled non-manual** | **Skilled manual** | **Partly skilled** | **Economically inactive** | **Total** |
| **Education** | 446 | 32 | 50 | 37 | 52 | 17 | 634 |
| **Empl, Professional** | 421 | 13 | 12 | 7 | 3 | 1 | 457 |
| **Empl Managerial** | 930 | 1,714 | 182 | 110 | 75 | 14 | 3025 |
| **Empl Skilled NM** | 349 | 81 | 2,088 | 90 | 65 | 41 | 2714 |
| **Empl Skilled M** | 64 | 51 | 62 | 1,841 | 218 | 11 | 2247 |
| **Empl Partly skilled** | 65 | 17 | 49 | 95 | 983 | 52 | 1261 |
| **Empl Unskilled** | 9 | 5 | 8 | 23 | 188 | 17 | 250 |
| **Inactive** | 59 | 47 | 110 | 71 | 85 | 845 | 1217 |
| **Unemployed** | 82 | 15 | 25 | 51 | 243 | 5 | 421 |
| **Missing** | 90 | 7 | 15 | 26 | 55 | 4 | 197 |
| **Total** | 2,515 | 1,982 | 2,601 | 2,351 | 1,967 | 1,007 | 12,423 |



Winpenny EM, *et al. J Epidemiol Community Health* 2021; 75:1172–1180. doi: 10.1136/jech-2021-216611

**Table S4: Estimated marginal means (with confidence intervals) of each outcome for each socioeconomic trajectory class**

| | **Waist circumference (cm)** | | **Systolic blood pressure (mmHg)** | | **Diastolic blood pressure (mmHg)** | |
|---|---|---|---|---|---|---|
| Socioeconomic trajectory class | Male (n=6,130) | Female (n=6,293) | Male (n=6,130) | Female (n=6,293) | Male (n=6,130) | Female (n=6,293) |
| Continued education | 99.3 (98.5, 100.1) | 88.5 (87.6, 89.5) | 128.9 (127.8, 130.0) | 119.6 (118.4, 120.9) | 79.3 (78.5, 80.1) | 75.2 (74.3, 76.0) |
| Managerial | 101.4 (100.5, 102.4) | 90.1 (89.0, 91.2) | 130.5 (129.4, 131.5) | 120.8 (119.6, 122.1) | 80.2 (79.4, 80.9) | 75.8 (74.9, 76.8) |
| Skilled non-manual | 101.7 (100.5, 102.8) | 90.5 (89.8, 91.3) | 131.0 (129.7, 132.3) | 121.4 (120.5, 122.3) | 80.3 (79.3, 81.3) | 76.2 (75.6, 76.8) |
| Skilled manual | 101.7 (100.9, 102.4) | 90.7 (89.4, 92.0) | 131.3 (130.4, 132.2) | 121.5 (119.8, 123.1) | 80.3 (79.7, 81.0) | 76.0 (74.9, 77.2) |
| Partly skilled | 102.2 (101.2, 103.2) | 92.8 (91.6, 93.9) | 130.9 (129.8, 132.1) | 122.2 (120.8, 123.5) | 80.1 (79.3, 81.0) | 76.4 (75.4, 77.4) |
| Economically inactive | 102.2 (98.9, 105.6) | 92.6 (91.4, 93.8) | 129.8 (125.6, 133.9) | 119.4 (117.9, 120.9) | 79.4 (76.1, 82.8) | 74.9 (73.8, 76.0) |
| Joint test | P<0.001 | P<0.001 | P=0.031 | P=0.012 | P=0.480 | P=0.108 |
| | **HDL cholesterol (mmol/L)** | | **Non-HDL cholesterol (mmol/L)** | | **Triglycerides (mmol/L)** | |
| Socioeconomic trajectory class | Male (n=6,130) | Female (n=6,293) | Male (n=6,130) | Female (n=6,293) | Male (n=6,130) | Female (n=6,293) |
| Continued education | 1.33 (1.30, 1.35) | 1.66 (1.63, 1.70) | 4.28 (4.19, 4.37) | 3.60 (3.51, 3.70) | 1.82 (1.72, 1.92) | 1.21 (1.16, 1.26) |
| Managerial | 1.28 (1.25, 1.30) | 1.62 (1.58, 1.65) | 4.40 (4.31, 4.49) | 3.72 (3.61, 3.83) | 2.01 (1.92, 2.10) | 1.29 (1.22, 1.37) |
| Skilled non-manual | 1.26 (1.22, 1.30) | 1.56 (1.53, 1.59) | 4.35 (4.24, 4.46) | 3.66 (3.59, 3.74) | 2.04 (1.90, 2.20) | 1.33 (1.27, 1.39) |
| Skilled manual | 1.31 (1.29, 1.34) | 1.64 (1.59, 1.69) | 4.37 (4.29, 4.45) | 3.67 (3.52, 3.83) | 2.02 (1.92, 2.12) | 1.32 (1.21, 1.45) |
| Partly skilled | 1.29 (1.26, 1.32) | 1.56 (1.52, 1.60) | 4.35 (4.26, 4.45) | 3.74 (3.62, 3.85) | 1.98 (1.87, 2.10) | 1.35 (1.26, 1.45) |
| Economically inactive | 1.20 (1.10, 1.31) | 1.49 (1.45, 1.53) | 4.54 (4.22, 4.85) | 3.80 (3.66, 3.93) | 2.18 (1.83, 2.60) | 1.41 (1.32, 1.52) |
| Joint test | P=0.011 | P<0.001 | P=0.418 | P=0.193 | P=0.022 | P=0.018 |
| | **HbA1c (mmol/mol)** | | **CRP (mg/L)** | | | |
| Socioeconomic trajectory class | Male (n=6,130) | Female (n=6,293) | Male (n=6,082) | Female (n=6,228) | | |
| Continued education | 37.6 (37.0, 38.3) | 36.1 (35.4, 36.9) | 0.93 (0.85, 1.02) | 0.99 (0.89, 1.10) | | |
| Managerial | 38.0 (37.1, 38.8) | 35.9 (35.2, 36.7) | 1.10 (0.99, 1.22) | 1.13 (1.03, 1.24) | | |
| Skilled non-manual | 37.6 (36.8, 38.4) | 36.2 (35.7, 36.7) | 1.17 (1.05, 1.31) | 1.23 (1.12, 1.34) | | |
| Skilled manual | 38.2 (37.7, 38.7) | 36.5 (35.6, 37.4) | 1.10 (1.03, 1.18) | 1.15 (1.03, 1.28) | | |
| Partly skilled | 37.8 (37.1, 38.6) | 36.4 (35.5, 37.2) | 1.15 (1.03, 1.28) | 1.23 (1.12, 1.35) | | |
| Economically inactive | 38.8 (36.2, 41.4) | 36.9 (35.9, 37.8) | 1.39 (1.06, 1.81) | 1.41 (1.24, 1.60) | | |
| Joint test | P=0.769 | P=0.628 | P=0.005 | P<0.001 | | |

**Table S5: Estimated marginal means (with confidence intervals) of each outcome for SEP at age 24y**

| | **Waist circumference (cm)** | | **Systolic blood pressure (mmHg)** | | **HDL cholesterol (mmol/L)** | |
|---|---|---|---|---|---|---|
| SEP at age 24y | Male (n=5,968) | Female (n=6,258) | Male (n=5,968) | Female (n=6,258) | Male (n=5,968) | Female (n=6,258) |
| Education | 99.3 (97.8, 100.8) | 88.0 (86.2, 89.8) | 129.0 (127.1, 131.0) | 118.5 (116.4, 120.6) | 1.33 (1.28, 1.37) | 1.66 (1.60, 1.72) |
| Empl, Professional | 99.0 (97.4, 100.5) | 89.1 (86.7, 91.4) | 128.4 (126.5, 130.3) | 120.4 (117.5, 123.3) | 1.33 (1.28, 1.38) | 1.68 (1.59, 1.78) |
| Empl Managerial | 101.0 (100.3, 101.8) | 89.8 (89.0, 90.6) | 130.5 (129.6, 131.4) | 120.2 (119.3, 121.2) | 1.30 (1.27, 1.32) | 1.62 (1.59, 1.65) |
| Empl Skilled NM | 101.6 (100.6, 102.6) | 90.3 (89.6, 91.0) | 130.4 (129.2, 131.5) | 121.2 (120.4, 122.0) | 1.28 (1.25, 1.31) | 1.57 (1.54, 1.60) |
| Empl Skilled M | 101.5 (100.8, 102.3) | 91.2 (89.8, 92.6) | 131.1 (130.3, 132.0) | 121.8 (120.0, 123.6) | 1.30 (1.28, 1.33) | 1.61 (1.56, 1.67) |
| Empl Partly skilled | 102.1 (100.9, 103.4) | 92.3 (91.1, 93.6) | 131.5 (130.0, 133.0) | 121.7 (120.0, 123.3) | 1.30 (1.25, 1.34) | 1.56 (1.52, 1.61) |
| Empl Unskilled | 102.0 (99.8, 104.2) | 90.4 (86.7, 94.2) | 131.0 (128.4, 133.6) | 123.0 (118.3, 127.7) | 1.30 (1.23, 1.38) | 1.55 (1.43, 1.68) |
| Inactive | 102.3 (99.8, 104.7) | 92.2 (91.1, 93.2) | 129.0 (126.0, 132.1) | 120.0 (118.6, 121.4) | 1.22 (1.14, 1.29) | 1.52 (1.48, 1.55) |
| Unemployed | 101.4 (99.5, 103.2) | 93.1 (90.5, 95.6) | 130.2 (127.7, 132.8) | 123.3 (120.4, 126.1) | 1.27 (1.22, 1.32) | 1.55 (1.46, 1.64) |
| Joint test | P=0.029 | P<0.001 | P=0.206 | P=0.085 | P=0.212 | P<0.001 |

| | **Triglycerides (mmol/L)** | | **CRP (mg/L)** | |
|---|---|---|---|---|
| SEP at age 24y | Male (n=5,968) | Female (n=6,258) | Male (n=5,920) | Female (n=6,193) |
| Education | 1.84 (1.69, 2.00) | 1.20 (1.10, 1.31) | 0.96 (0.82, 1.13) | 1.01 (0.85, 1.19) |
| Empl, Professional | 1.76 (1.62, 1.92) | 1.20 (1.08, 1.33) | 0.92 (0.79, 1.07) | 1.07 (0.87, 1.33) |
| Empl Managerial | 1.96 (1.88, 2.04) | 1.26 (1.21, 1.31) | 1.05 (0.97, 1.15) | 1.10 (1.02, 1.19) |
| Empl Skilled NM | 1.99 (1.87, 2.11) | 1.32 (1.27, 1.38) | 1.13 (1.03, 1.25) | 1.20 (1.10, 1.30) |
| Empl Skilled M | 2.01 (1.93, 2.09) | 1.33 (1.21, 1.46) | 1.10 (1.03, 1.17) | 1.16 (1.04, 1.29) |
| Empl Partly skilled | 1.96 (1.85, 2.07) | 1.34 (1.25, 1.43) | 1.13 (1.00, 1.28) | 1.22 (1.10, 1.36) |
| Empl Unskilled | 1.97 (1.74, 2.23) | 1.41 (1.19, 1.67) | 1.13 (0.93, 1.37) | 1.27 (0.95, 1.69) |
| Inactive | 2.16 (1.88, 2.48) | 1.39 (1.32, 1.46) | 1.34 (1.06, 1.68) | 1.32 (1.19, 1.46) |
| Unemployed | 2.09 (1.92, 2.28) | 1.30 (1.17, 1.46) | 1.15 (0.97, 1.36) | 1.19 (0.98, 1.45) |
| Joint test | P=0.095 | P=0.036 | P=0.134 | P=0.076 |

**Table S6: Direct and indirect effects for models mediated by NS-SEC and household income at age 46.**

| | **Waist circumference** | | **Systolic blood pressure** | | **HDL cholesterol (% change)** | | **Triglycerides (% change)** | | **CRP (% change)** | |
|---|---|---|---|---|---|---|---|---|---|---|
| | Male (n=6,130) | Female (n=6,293) | Male (n=6,130) | Female (n=6,293) | Male (n=6,130) | Female (n=6,293) | Male (n=6,130) | Female (n=6,293) | Male (n=6,082) | Female (n=6,228) |
| **Direct effect of EA Socioeconomic trajectory class on outcome** | | | | | | | | | | |
| Managerial | 2.06 (0.80, 3.33) | 1.40 (-0.20, 2.99) | 1.51 (-0.04, 3.06) | 1.13 (-0.60, 2.85) | -3.25 (-5.92, -0.50) | -2.27 (-4.97, 0.60) | 9.75 (3.15, 16.88) | 5.76 (-1.88, 14.00) | 16.65 (2.02, 33.51) | 13.31 (-1.78, 30.73) |
| Skilled non-manual | 2.27 (0.80, 3.74) | 1.71 (0.39, 3.04) | 2.12 (0.42, 3.82) | 1.68 (0.05, 3.30) | -4.30 (-7.50, -1.00) | -5.45 (-8.15, -2.76) | 11.18 (2.63, 20.56) | 8.44 (1.82, 15.60) | 23.61 (9.09, 40.07) | 23.49 (7.57, 41.62) |
| Skilled manual | 2.07 (0.79, 3.35) | 1.63 (-0.14, 3.40) | 2.36 (0.63, 4.08) | 1.61 (-0.47, 3.70) | 0.40 (-2.47, 3.36) | -0.40 (-4.11, 3.46) | 8.65 (1.11, 16.77) | 6.93 (-3.92, 19.01) | 14.22 (0.30, 30.08) | 14.45 (-2.27, 34.04) |
| Partly skilled | 2.58 (1.16, 4.01) | 3.64 (1.89, 5.39) | 1.99 (0.24, 3.74) | 2.34 (0.27, 4.41) | -1.00 (-4.11, 2.22) | -4.78 (-8.24, -1.29) | 6.40 (-1.00, 14.34) | 8.65 (-0.10, 18.06) | 18.18 (0.40, 39.24) | 22.26 (3.56, 44.34) |
| Economically inactive | 2.67 (-0.61, 5.94) | 3.57 (1.94, 5.19) | 0.72 (-3.71, 5.14) | -0.51 (-2.67, 1.66) | -7.87 (-15.63, 0.50) | -9.24 (-12.45, -6.01) | 17.23 (-0.90, 38.68) | 13.88 (4.08, 24.61) | 43.33 (9.64, 87.39) | 40.49 (18.29, 66.86) |
| NS-SEC | 0.26 (-0.15, 0.67) | 0.42 (-0.18, 1.01) | -0.01 (-0.45, 0.43) | 0.08 (-0.50, 0.65) | -1.09 (-1.98, -0.10) | -0.90 (-1.78, 0.00) | 1.51 (-0.60, 3.67) | 2.02 (-0.10, 4.19) | 2.63 (-2.37, 7.79) | 0.90 (-3.92, 6.08) |
| Income[1] | -0.01 (-0.06, 0.05) | -0.01 (-0.05, 0.03) | -0.03 (-0.08, 0.03) | -0.05 (-0.09, 0.00) | 0.10 (0.00, 0.20) | 0.10 (0.00, 0.20) | -0.20 (-0.40, 0.10) | -0.10 (-0.30, 0.00) | -0.10 (-0.60, 0.30) | -0.10 (-0.50, 0.30) |
| **Indirect effect via NS-SEC8 (1)** | | | | | | | | | | |
| Managerial | 0.10 (-0.06, 0.26) | 0.15 (-0.07, 0.38) | 0.00 (-0.17, 0.17) | 0.03 (-0.18, 0.24) | -0.40 (-0.80, 0.00) | -0.30 (-0.70, 0.00) | 0.60 (-0.30, 1.41) | 0.70 (-0.10, 1.61) | 1.01 (-1.00, 3.05) | 0.40 (-1.49, 2.22) |
| Skilled non-manual | 0.14 (-0.08, 0.35) | 0.24 (-0.11, 0.59) | 0.00 (-0.23, 0.23) | 0.05 (-0.28, 0.37) | -0.60 (-1.09, 0.00) | -0.50 (-1.09, 0.00) | 0.80 (-0.40, 1.92) | 1.11 (-0.10, 2.43) | 1.41 (-1.29, 4.19) | 0.60 (-2.27, 3.46) |
| Skilled manual | 0.31 (-0.17, 0.79) | 0.47 (-0.20, 1.14) | -0.01 (-0.53, 0.51) | 0.09 (-0.55, 0.72) | -1.29 (-2.37, -0.10) | -1.00 (-2.08, 0.00) | 1.71 (-0.80, 4.29) | 2.22 (-0.20, 4.71) | 3.15 (-2.86, 9.42) | 1.11 (-4.40, 6.82) |
| Partly skilled | 0.35 (-0.20, 0.90) | 0.52 (-0.22, 1.27) | -0.01 (-0.60, 0.58) | 0.09 (-0.62, 0.81) | -1.39 (-2.66, -0.10) | -1.09 (-2.27, 0.00) | 2.02 (-0.90, 4.92) | 2.53 (-0.20, 5.34) | 3.46 (-3.15, 10.52) | 1.21 (-4.88, 7.57) |
| Economically inactive | 0.31 (-0.18, 0.79) | 0.47 (-0.20, 1.14) | -0.01 (-0.54, 0.51) | 0.09 (-0.56, 0.73) | -1.29 (-2.47, -0.10) | -1.00 (-2.08, 0.00) | 1.71 (-0.80, 4.29) | 2.22 (-0.10, 4.71) | 3.05 (-2.86, 9.20) | 1.11 (-4.50, 6.93) |
| **Indirect effect via equivalised household income (2)** | | | | | | | | | | |
| Managerial | 0.00 (-0.03, 0.04) | 0.00 (-0.03, 0.02) | 0.01 (-0.04, 0.06) | 0.00 (-0.07, 0.07) | -0.10 (-0.20, 0.10) | 0.00 (-0.20, 0.20) | 0.10 (-0.20, 0.30) | 0.00 (-0.30, 0.20) | 0.10 (-0.30, 0.40) | 0.00 (-0.30, 0.40) |
| Skilled non-manual | 0.00 (-0.04, 0.05) | 0.00 (-0.03, 0.03) | 0.02 (-0.04, 0.07) | 0.00 (-0.07, 0.07) | -0.10 (-0.20, 0.10) | 0.00 (-0.20, 0.20) | 0.10 (-0.20, 0.40) | 0.00 (-0.20, 0.30) | 0.10 (-0.30, 0.50) | 0.00 (-0.30, 0.30) |
| Skilled manual | 0.00 (-0.03, 0.03) | -0.02 (-0.09, 0.05) | 0.00 (-0.04, 0.04) | -0.07 (-0.19, 0.06) | 0.00 (-0.10, 0.10) | 0.20 (-0.10, 0.40) | 0.00 (-0.20, 0.20) | -0.20 (-0.70, 0.20) | 0.00 (-0.30, 0.30) | -0.20 (-0.90, 0.50) |
| Partly skilled | 0.01 (-0.04, 0.05) | -0.01 (-0.06, 0.04) | 0.02 (-0.04, 0.08) | -0.04 (-0.15, 0.06) | -0.10 (-0.20, 0.10) | 0.10 (-0.10, 0.30) | 0.10 (-0.20, 0.40) | -0.10 (-0.50, 0.20) | 0.10 (-0.30, 0.50) | -0.10 (-0.70, 0.50) |



Winpenny EM, *et al. J Epidemiol Community Health* 2021; 75:1172–1180. doi: 10.1136/jech-2021-216611

| | | | | | | | | | | |
|---|---|---|---|---|---|---|---|---|---|---|
| Economically inactive | 0.01 (-0.09, 0.12) | 0.00 (-0.04, 0.04) | 0.05 (-0.09, 0.18) | -0.01 (-0.11, 0.08) | -0.20 (-0.60, 0.20) | 0.00 (-0.20, 0.30) | 0.30 (-0.40, 1.11) | 0.00 (-0.40, 0.30) | 0.20 (-0.70, 1.21) | 0.00 (-0.40, 0.40) |

Note: (1) The National Statistics Socio-economic classification (8 classes), (2) Equivalised household income per week (in £100s)

**Table S7: Cross-sectional associations between NS-SEC, equivalised household income and metabolic outcomes at age 46.**

| | **Unadjusted** | | **Adjusted for childhood covariates** | | **Adjusted for childhood covariates and early adulthood socioeconomic trajectory class** | |
|---|---|---|---|---|---|---|
| | **Waist circumference (cm)** | | | | | |
| NS-SEC, Ref: Higher managerial and administrative occupations | Male (n=6,130) | Female (n=6,293) | Male (n=6,130) | Female (n=6,092) | Male (n=6,130) | Female (n=6,293) |
| Lower managerial and administrative | 1.73 (0.63, 2.83) | 1.63 (0.25, 3.01) | 1.07 (-0.03, 2.18) | 0.86 (-0.51, 2.22) | 0.69 (-0.44, 1.82) | 0.52 (-0.87, 1.91) |
| Intermediate occupations | 2.71 (1.00, 4.42) | 2.33 (0.66, 4.00) | 1.58 (0.01, 3.16) | 1.33 (-0.24, 2.89) | 0.90 (-0.75, 2.55) | 0.69 (-0.93, 2.30) |
| Small employers and own account workers | 2.25 (0.81, 3.69) | 2.70 (0.59, 4.82) | 0.89 (-0.54, 2.32) | 1.46 (-0.61, 3.53) | 0.14 (-1.37, 1.64) | 0.70 (-1.41, 2.82) |
| Lower supervisory and technical | 2.49 (1.00, 3.99) | 4.25 (1.89, 6.61) | 1.11 (-0.40, 2.62) | 2.44 (0.14, 4.74) | 0.27 (-1.40, 1.93) | 1.45 (-0.97, 3.87) |
| Semi-routine occupations | 3.22 (1.55, 4.89) | 4.66 (2.78, 6.54) | 1.76 (0.13, 3.39) | 2.73 (0.81, 4.65) | 0.88 (-0.76, 2.51) | 1.61 (-0.47, 3.69) |
| Routine occupations | 3.94 (2.38, 5.51) | 4.68 (2.17, 7.19) | 2.49 (0.89, 4.08) | 2.79 (0.35, 5.22) | 1.57 (-0.21, 3.34) | 1.57 (-1.04, 4.18) |
| Never worked and long-term unemployed | 2.54 (-0.64, 5.72) | 4.46 (0.89, 8.04) | 1.37 (-1.79, 4.54) | 2.54 (-1.06, 6.14) | 0.61 (-2.51, 3.74) | 1.41 (-2.34, 5.17) |
| Joint test of NS-SEC | P<0.001 | P<0.001 | P=0.16 | P=0.05 | P=0.71 | P=0.76 |
| Equivalised household income (£100s/week) | -0.05 (-0.10, 0.01) | -0.07 (-0.11, -0.02) | -0.02 (-0.07, 0.04) | -0.03 (-0.07, 0.01) | -0.01 (-0.06, 0.04) | -0.02 (-0.06, 0.02) |
| | **Systolic blood pressure (mmHg)** | | | | | |
| | Male (n=6,130) | Female (n=6,293) | Male (n=6,130) | Female (n=6,293) | Male (n=6,130) | Female (n=6,293) |
| Lower managerial and administrative | 0.57 (-0.71, 1.86) | 0.85 (-0.67, 2.37) | 0.25 (-1.03, 1.53) | 0.46 (-1.12, 2.03) | -0.09 (-1.40, 1.21) | 0.33 (-1.26, 1.92) |
| Intermediate occupations | 1.14 (-0.96, 3.25) | 1.56 (-0.18, 3.29) | 0.62 (-1.45, 2.69) | 0.86 (-0.92, 2.65) | -0.01 (-2.09, 2.08) | 0.51 (-1.34, 2.36) |
| Small employers and own account workers | 0.90 (-0.74, 2.55) | 1.26 (-0.71, 3.24) | 0.28 (-1.33, 1.89) | 0.53 (-1.52, 2.57) | -0.46 (-2.16, 1.24) | 0.26 (-1.84, 2.37) |
| Lower supervisory and technical | 1.42 (-0.13, 2.97) | 2.82 (0.47, 5.16) | 0.73 (-0.84, 2.30) | 1.77 (-0.57, 4.11) | -0.10 (-1.74, 1.53) | 1.50 (-0.95, 3.96) |
| Semi-routine occupations | 1.45 (-0.50, 3.39) | 1.76 (0.08, 3.45) | 0.67 (-1.29, 2.63) | 0.65 (-1.15, 2.44) | -0.10 (-2.22, 2.02) | 0.36 (-1.53, 2.25) |
| Routine occupations | 2.61 (0.69, 4.54) | 2.01 (-0.46, 4.48) | 1.85 (-0.11, 3.81) | 0.82 (-1.70, 3.34) | 0.99 (-1.03, 3.02) | 0.53 (-2.16, 3.22) |



Winpenny EM, *et al. J Epidemiol Community Health* 2021; 75:1172–1180. doi: 10.1136/jech-2021-216611

| | | | | | | |
|---|---|---|---|---|---|---|
| Never worked and long-term unemployed | -0.05 (-3.01, 2.91) | 2.56 (-0.97, 6.08) | -0.60 (-3.48, 2.28) | 1.50 (-1.97, 4.97) | -1.28 (-4.21, 1.66) | 1.28 (-2.27, 4.83) |
| Joint test of NS-SEC | P=0.24 | P=0.22 | P=0.72 | P=0.88 | P=0.92 | P=0.95 |
| Equivalised household income (£100s/week) | -0.04 (-0.10, 0.01) | -0.07 (-0.11, -0.02) | -0.03 (-0.09, 0.03) | -0.05 (-0.09, -0.01) | -0.03 (-0.09, 0.03) | -0.05 (-0.09, -0.01) |
| | **Diastolic blood pressure (mmHg)** | | | | | |
| | Male (n=6,130) | Female (n=6,293) | Male (n=6,130) | Female (n=6,293) | Male (n=6,130) | Female (n=6,293) |
| Lower managerial and administrative | 0.32 (-0.71, 1.35) | 0.20 (-0.88, 1.29) | 0.02 (-1.00, 1.05) | -0.11 (-1.21, 0.99) | -0.17 (-1.23, 0.89) | -0.20 (-1.30, 0.90) |
| Intermediate occupations | 0.84 (-0.77, 2.45) | 0.55 (-0.64, 1.74) | 0.38 (-1.21, 1.96) | 0.01 (-1.20, 1.22) | 0.03 (-1.59, 1.66) | -0.23 (-1.44, 0.98) |
| Small employers and own account workers | -0.35 (-1.54, 0.84) | 0.25 (-1.16, 1.66) | -0.89 (-2.08, 0.30) | -0.37 (-1.81, 1.07) | -1.32 (-2.57, -0.06) | -0.54 (-2.01, 0.92) |
| Lower supervisory and technical | 0.39 (-0.79, 1.56) | 1.05 (-0.53, 2.64) | -0.24 (-1.48, 1.00) | 0.19 (-1.38, 1.77) | -0.71 (-2.02, 0.60) | 0.01 (-1.58, 1.60) |
| Semi-routine occupations | 0.38 (-1.15, 1.91) | 0.59 (-0.64, 1.81) | -0.30 (-1.88, 1.28) | -0.35 (-1.64, 0.94) | -0.74 (-2.42, 0.94) | -0.55 (-1.87, 0.78) |
| Routine occupations | 1.22 (-0.19, 2.63) | 0.33 (-1.44, 2.11) | 0.55 (-0.93, 2.02) | -0.65 (-2.43, 1.14) | 0.06 (-1.49, 1.61) | -0.85 (-2.67, 0.98) |
| Never worked and long-term unemployed | -0.09 (-2.58, 2.40) | 1.55 (-1.16, 4.26) | -0.59 (-2.99, 1.81) | 0.63 (-2.10, 3.36) | -0.96 (-3.45, 1.52) | 0.48 (-2.31, 3.26) |
| Joint test of NS-SEC | P=0.64 | P=0.84 | P=0.72 | P=0.97 | P=0.55 | P=0.95 |
| Equivalised household income (£100s/week) | -0.01 (-0.05, 0.04) | -0.03 (-0.06, 0.00) | 0.00 (-0.05, 0.05) | -0.02 (-0.05, 0.01) | 0.00 (-0.04, 0.05) | -0.02 (-0.05, 0.01) |
| | **HDL cholesterol (% change)** | | | | | |
| | Male (n=6,130) | Female (n=6,293) | Male (n=6,130) | Female (n=6,293) | Male (n=6,130) | Female (n=6,293) |
| Lower managerial and administrative | -3.72 (-6.27, -1.11) | -3.90 (-6.49, -1.24) | -2.63 (-5.21, 0.01) | -2.89 (-5.43, -0.27) | -2.35 (-4.96, 0.34) | -2.27 (-4.84, 0.37) |
| Intermediate occupations | -5.64 (-8.89, -2.27) | -6.47 (-9.20, -3.66) | -3.73 (-7.03, -0.30) | -4.94 (-7.73, -2.07) | -3.40 (-6.80, 0.12) | -3.46 (-6.37, -0.46) |
| Small employers and own account workers | -3.35 (-6.43, -0.16) | -4.58 (-8.30, -0.70) | -1.12 (-4.21, 2.07) | -2.72 (-6.40, 1.10) | -1.30 (-4.56, 2.08) | -1.34 (-5.11, 2.59) |
| Lower supervisory and technical | -4.14 (-7.38, -0.80) | -7.66 (-11.76, -3.36) | -1.67 (-5.06, 1.83) | -4.86 (-9.19, -0.33) | -1.95 (-5.52, 1.75) | -3.35 (-7.77, 1.29) |
| Semi-routine occupations | -5.91 (-9.37, -2.32) | -8.99 (-11.86, -6.02) | -3.32 (-6.98, 0.48) | -6.20 (-9.22, -3.09) | -3.39 (-7.21, 0.60) | -4.43 (-7.69, -1.05) |
| Routine occupations | -7.32 (-10.97, -3.52) | -9.66 (-13.34, -5.82) | -4.61 (-8.35, -0.72) | -6.70 (-10.48, -2.76) | -4.84 (-8.60, -0.92) | -5.00 (-8.85, -0.98) |

| | | | | | | |
|---|---|---|---|---|---|---|
| Never worked and long-term unemployed | -8.53 (-14.52, -2.13) | -9.47 (-14.86, -3.74) | -6.25 (-12.28, 0.20) | -6.36 (-11.81, -0.56) | -6.28 (-12.16, -0.01) | -4.69 (-10.37, 1.35) |
| Joint test of NS-SEC | P<0.001 | P<0.001 | P=0.11 | P=0.003 | P=0.16 | P=0.17 |
| Equivalised household income (£100s/week) | 0.19 (0.06, 0.31) | 0.20 (0.11, 0.28) | 0.14 (0.02, 0.26) | 0.15 (0.06, 0.24) | 0.13 (0.02, 0.25) | 0.13 (0.04, 0.22) |
| | **Non-HDL cholesterol (mmol/L)** | | | | | |
| | Male (n=6,130) | Female (n=6,293) | Male (n=6,130) | Female (n=6,293) | Male (n=6,130) | Female (n=6,293) |
| Lower managerial and administrative | 0.09 (-0.01, 0.19) | 0.09 (-0.02, 0.21) | 0.07 (-0.03, 0.17) | 0.07 (-0.05, 0.19) | 0.05 (-0.05, 0.15) | 0.06 (-0.06, 0.18) |
| Intermediate occupations | 0.14 (-0.01, 0.30) | 0.13 (0.00, 0.26) | 0.11 (-0.05, 0.26) | 0.09 (-0.05, 0.22) | 0.09 (-0.07, 0.25) | 0.07 (-0.06, 0.20) |
| Small employers and own account workers | 0.11 (-0.03, 0.24) | 0.15 (-0.01, 0.31) | 0.07 (-0.07, 0.20) | 0.10 (-0.06, 0.25) | 0.05 (-0.09, 0.19) | 0.08 (-0.08, 0.23) |
| Lower supervisory and technical | 0.09 (-0.04, 0.23) | 0.23 (0.05, 0.42) | 0.05 (-0.10, 0.19) | 0.16 (-0.03, 0.34) | 0.03 (-0.12, 0.18) | 0.13 (-0.06, 0.32) |
| Semi-routine occupations | 0.18 (0.03, 0.34) | 0.22 (0.05, 0.40) | 0.13 (-0.03, 0.29) | 0.14 (-0.04, 0.32) | 0.11 (-0.05, 0.28) | 0.11 (-0.06, 0.28) |
| Routine occupations | 0.11 (-0.05, 0.28) | 0.25 (0.04, 0.45) | 0.06 (-0.10, 0.22) | 0.16 (-0.06, 0.38) | 0.04 (-0.13, 0.21) | 0.13 (-0.09, 0.35) |
| Never worked and long-term unemployed | 0.17 (-0.15, 0.48) | 0.09 (-0.02, 0.21) | 0.12 (-0.19, 0.42) | 0.20 (-0.08, 0.49) | 0.11 (-0.20, 0.41) | 0.17 (-0.10, 0.45) |
| Joint test of NS-SEC | P=0.42 | P=0.03 | P=0.84 | P=0.53 | P=0.91 | P=0.83 |
| Equivalised household income (£100s/week) | 0.00 (-0.01, 0.00) | 0.00 (-0.01, 0.00) | 0.00 (-0.01, 0.00) | 0.00 (-0.01, 0.00) | 0.00 (-0.01, 0.00) | 0.00 (-0.01, 0.00) |
| | **Triglycerides (% change)** | | | | | |
| | Male (n=6,130) | Female (n=6,293) | Male (n=6,130) | Female (n=6,293) | Male (n=6,130) | Female (n=6,293) |
| Lower managerial and administrative | 6.14 (-0.21, 12.89) | 7.43 (0.32, 15.05) | 4.72 (-1.57, 11.42) | 5.89 (-1.36, 13.69) | 3.15 (-3.08, 9.79) | 4.76 (-2.26, 12.28) |
| Intermediate occupations | 15.28 (6.17, 25.18) | 14.79 (7.06, 23.07) | 12.60 (3.47, 22.53) | 12.46 (4.85, 20.62) | 9.87 (1.01, 19.51) | 9.88 (2.66, 17.60) |
| Small employers and own account workers | 4.88 (-4.62, 15.33) | 7.18 (-2.63, 17.96) | 2.28 (-7.44, 13.01) | 4.63 (-4.87, 15.08) | -0.24 (-9.90, 10.46) | 2.21 (-6.79, 12.08) |
| Lower supervisory and technical | 8.01 (-0.36, 17.09) | 13.84 (3.15, 25.64) | 5.17 (-3.68, 14.84) | 9.59 (-0.77, 21.02) | 2.32 (-6.98, 12.55) | 6.47 (-4.05, 18.14) |
| Semi-routine occupations | 14.75 (4.73, 25.73) | 18.52 (9.37, 28.45) | 11.42 (1.26, 22.60) | 14.20 (5.65, 23.46) | 8.72 (-1.19, 19.64) | 10.67 (1.80, 20.30) |
| Routine occupations | 9.00 (0.16, 18.63) | 14.23 (2.22, 27.65) | 5.83 (-2.68, 15.08) | 9.80 (-2.43, 23.55) | 3.04 (-5.55, 12.40) | 6.21 (-5.29, 19.10) |

| | | | | | | |
|---|---|---|---|---|---|---|
| Never worked and long-term unemployed | 18.42 (1.78, 37.79) | 24.64 (8.55, 43.12) | 15.20 (-0.44, 33.29) | 19.16 (4.22, 36.23) | 12.87 (-2.67, 30.88) | 15.76 (0.63, 33.18) |
| Joint test of NS-SEC | P=0.02 | P=0.002 | P=0.15 | P=0.05 | P=0.34 | P=0.28 |
| Equivalised household income (£100s/week) | -0.27 (-0.56, 0.02) | -0.28 (-0.48, -0.08) | -0.21 (-0.49, 0.07) | -0.22 (-0.41, -0.03) | -0.19 (-0.47, 0.09) | -0.19 (-0.38, 0.00) |
| | **HbA1c (mmol/mol)** | | | | | |
| | Male (n=6,130) | Female (n=6,293) | Male (n=6,130) | Female (n=6,293) | Male (n=6,130) | Female (n=6,293) |
| Lower managerial and administrative | 0.54 (-0.26, 1.34) | 0.31 (-0.77, 1.39) | 0.30 (-0.51, 1.12) | 0.14 (-0.94, 1.21) | 0.33 (-0.50, 1.16) | 0.14 (-0.96, 1.24) |
| Intermediate occupations | 1.38 (0.10, 2.66) | 1.14 (0.14, 2.15) | 0.96 (-0.33, 2.24) | 0.89 (-0.18, 1.96) | 1.06 (-0.31, 2.42) | 0.92 (-0.16, 2.00) |
| Small employers and own account workers | 1.46 (0.43, 2.50) | 1.41 (0.07, 2.75) | 0.97 (-0.07, 2.02) | 1.06 (-0.28, 2.40) | 1.08 (0.02, 2.15) | 1.05 (-0.35, 2.45) |
| Lower supervisory and technical | 0.73 (-0.47, 1.94) | 1.66 (0.00, 3.33) | 0.20 (-1.07, 1.46) | 1.14 (-0.46, 2.73) | 0.33 (-0.93, 1.60) | 1.09 (-0.53, 2.72) |
| Semi-routine occupations | 1.67 (0.53, 2.82) | 1.76 (0.66, 2.87) | 1.13 (-0.04, 2.29) | 1.24 (0.03, 2.45) | 1.30 (0.11, 2.49) | 1.19 (-0.12, 2.49) |
| Routine occupations | 2.37 (1.14, 3.61) | 1.89 (0.47, 3.32) | 1.78 (0.58, 2.97) | 1.31 (-0.21, 2.84) | 1.95 (0.72, 3.19) | 1.24 (-0.44, 2.92) |
| Never worked and long-term unemployed | 0.98 (-1.64, 3.59) | 1.23 (-1.10, 3.56) | 0.44 (-2.15, 3.03) | 0.63 (-1.74, 3.00) | 0.61 (-1.97, 3.19) | 0.54 (-1.87, 2.95) |
| Joint test of NS-SEC | P=0.01 | P=0.01 | P=0.19 | P=0.20 | P=0.15 | P=0.34 |
| Equivalised household income (£100s/week) | -0.04 (-0.07, 0.00) | -0.03 (-0.06, -0.01) | -0.03 (-0.06, 0.01) | -0.02 (-0.05, 0.00) | -0.03 (-0.06, 0.01) | -0.02 (-0.05, 0.00) |
| | **CRP (% change)** | | | | | |
| | Male (n=6,082) | Female (n=6,228) | Male (n=6,082) | Female (n=6,228) | Male (n=6,082) | Female (n=6,228) |
| Lower managerial and administrative | 16.0 (4.50, 28.7) | 12.2 (-0.55, 26.5) | 12.57 (1.26, 25.14) | 8.12 (-3.82, 21.54) | 9.51 (-1.82, 22.15) | 5.39 (-6.44, 18.72) |
| Intermediate occupations | 15.0 (-3.72, 37.3) | 14.6 (-0.72, 32.3) | 9.05 (-8.90, 30.54) | 8.63 (-5.82, 25.28) | 3.67 (-13.36, 24.06) | 2.25 (-11.76, 18.48) |
| Small employers and own account workers | 22.4 (7.53, 39.4) | 20.2 (1.19, 42.8) | 15.83 (1.07, 32.76) | 12.70 (-4.89, 33.53) | 10.55 (-5.41, 29.21) | 6.41 (-11.03, 27.27) |
| Lower supervisory and technical | 13.3 (-1.94, 30.9) | 17.6 (-2.93, 42.4) | 5.94 (-8.94, 23.27) | 8.12 (-11.15, 31.57) | 0.52 (-15.61, 19.74) | 0.91 (-18.60, 25.10) |
| Semi-routine occupations | 26.4 (5.99, 50.7) | 22.9 (3.10, 46.4) | 17.35 (-3.24, 42.31) | 11.86 (-6.37, 33.64) | 10.77 (-10.67, 37.35) | 3.54 (-14.87, 25.94) |
| Routine occupations | 28.5 (9.85, 50.4) | 28.1 (8.25, 51.5) | 19.23 (0.40, 41.58) | 16.21 (-2.79, 38.92) | 12.57 (-7.94, 37.64) | 7.28 (-12.02, 30.82) |

| | | | | | | |
|---|---|---|---|---|---|---|
| Never worked and long-term unemployed | 32.1 (-8.85, 91.5) | 32.7 (-9.92, 95.5) | 23.98 (-14.22, 79.20) | 20.19 (-19.28, 78.96) | 17.82 (-17.92, 69.13) | 11.99 (-24.75, 66.67) |
| Joint test of NS-SEC | P=0.04 | P=0.17 | P=0.38 | P=0.84 | P=0.73 | P=0.99 |
| Equivalised household income (£100s/week) | -0.35 (-0.82, 0.12) | -0.34 (-0.74, 0.05) | -0.23 (-0.70, 0.25) | -0.20 (-0.63, 0.23) | -0.16 (-0.64, 0.31) | -0.15 (-0.58, 0.29) |

Note: NS-SEC: UK National Statistics Socio-economic Classification

BMJ Publishing Group Limited (BMJ) disclaims all liability and responsibility arising from any reliance placed on this supplemental material which has been supplied by the author(s)



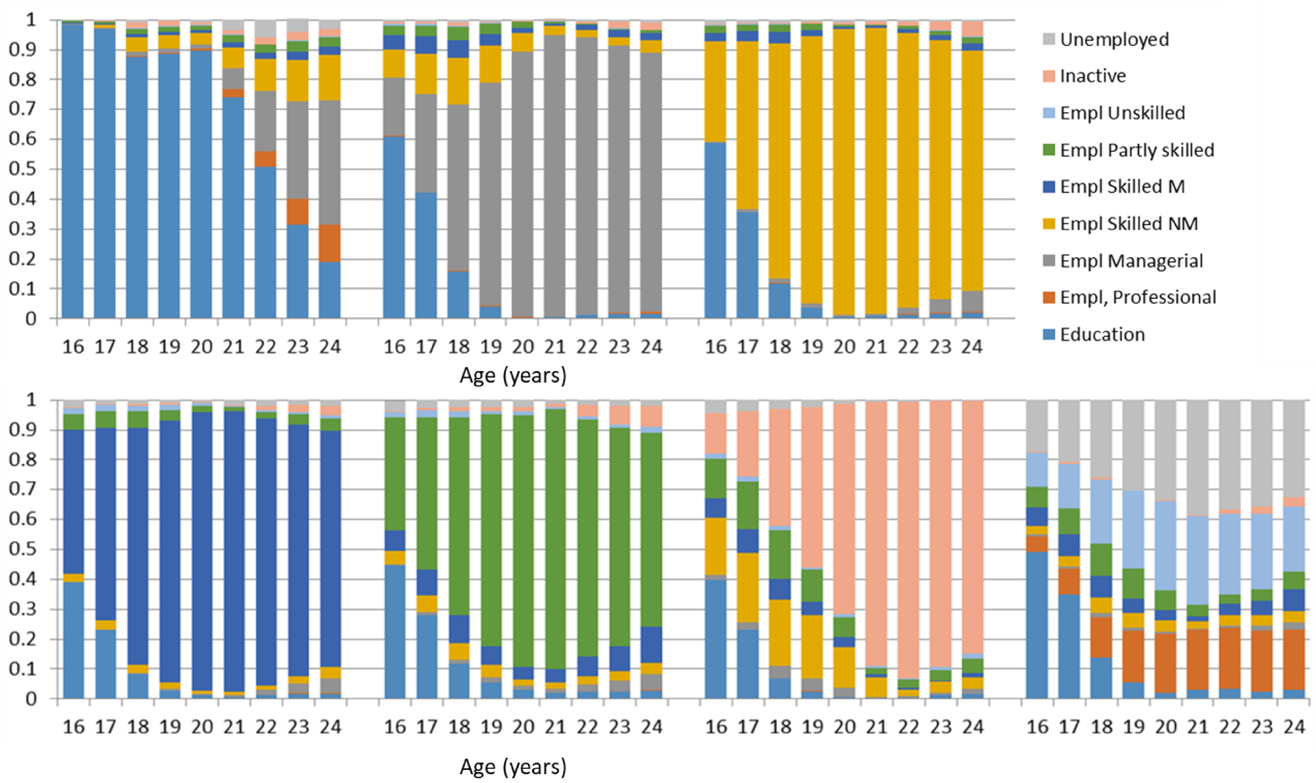


**Figure S8: The seven class LCA solution showing, for each class, response probabilities for participation in different economic activities at each year of age**
